# Salinity Affects Saxitoxins (STXs) Toxicity in the Dinoflagellate *Alexandrium pacificum*, with Low Transcription of SXT-Biosynthesis Genes *sxtA4* and *sxtG*

**DOI:** 10.3390/toxins13100733

**Published:** 2021-10-18

**Authors:** Quynh Thi Nhu Bui, Hansol Kim, Hyunjun Park, Jang-Seu Ki

**Affiliations:** Department of Biotechnology, Sangmyung University, Seoul 03016, Korea; 202033036@sangmyung.kr (Q.T.N.B.); 201934001@sangmyung.kr (H.K.); 202032006@sangmyung.kr (H.P.)

**Keywords:** dinoflagellate, *Alexandrium pacificum*, salinity, toxin production, saxitoxin biosynthesis gene

## Abstract

Salinity is an important factor for regulating metabolic processes in aquatic organisms; however, its effects on toxicity and STX biosynthesis gene responses in dinoflagellates require further elucidation. Herein, we evaluated the physiological responses, toxin production, and expression levels of two STX synthesis core genes, *sxtA4* and *sxtG*, in the dinoflagellate *Alexandrium pacificum* Alex05 under different salinities (20, 25, 30, 35, and 40 psu). Optimal growth was observed at 30 psu (0.12 cell division/d), but cell growth significantly decreased at 20 psu and was irregular at 25 and 40 psu. The cell size increased at lower salinities, with the highest size of 31.5 µm at 20 psu. STXs eq was highest (35.8 fmol/cell) in the exponential phase at 30 psu. GTX4 and C2 were predominant at that time but were replaced by GTX1 and NeoSTX in the stationary phase. However, *sxtA4* and *sxtG* mRNAs were induced, and their patterns were similar in all tested conditions. PCA showed that gene transcriptional levels were not correlated with toxin contents and salinity. These results suggest that *A. pacificum* may produce the highest amount of toxins at optimal salinity, but *sxtA4* and *sxtG* may be only minimally affected by salinity, even under high salinity stress.

## 1. Introduction

The dinoflagellate *Alexandrium* is an armoured photosynthetic microeukaryote that is distributed in coastal waters [1,2]. To date, more than 34 species have been described morphologically [3]. Some grow rapidly when growth conditions are optimal, causing harmful algal blooms or red tides [4]. In addition, they can produce potent biotoxins, such as saxitoxin analogues (STXs), spiroimines, goniodomins, and lytic compounds [4,5,6]. These toxins can cause paralytic shellfish poisoning (PSP) when toxin-contaminated shellfish are consumed [7]. More than 2000 cases of PSP were recorded around the world in the 2000s, and this issue poses a global threat to human health [8,9].

The STXs are one of the most potent neurotoxins and were first reported in butter clam and mussel tissues in 1957 [10]. They are produced naturally by the toxic dinoflagellates *Alexandrium* spp., *Gymnodinium catenatum*, and *Pyrodinium bahamense* in marine environments [11,12,13] and the freshwater cyanobacteria *Aphanizomenon gracile*, *Cylindrospermopsis raciborskii*, and *Dolichospermum* spp. [14,15,16]. Based on the position of the side group moieties (R1–R4) on the carbon backbone, structural families of STX are classified into non-sulphated (STX and Neosaxitoxin [NeoSTX]), mono-sulphated (Gonyautoxins [GTXs]), di-sulphated (N-sulfocarbamoyl toxins, C1–4), decarbamoylated (dc-toxins), and deoxy-decarbamoylated (do-toxins) [17,18]. To date, at least 57 natural analogues of STX have been identified [19].

Environmental conditions are important factors in phytoplankton cell growth [20,21]. These include nutrients, temperature, light, and/or salinity, and possibly influence the STX production in *Alexandrium* [22,23,24]. The cellular toxin quota of *Alexandrium minutum*, for example, was much higher when cultured under phosphate-limitation, but it decreased abruptly in phosphate-replete conditions [25]. In addition, temperature and light similarly affected cell growth in *Alexandrium*, and differences in cell toxicity were observed [21,26]. Thus, environmental factors affect the physiological metabolism of *Alexandrium* cells and thus are expected to modulate the biosynthesis mechanisms of intracellular toxins.

Salinity is an important environmental factor that regulates the physiological processes of aquatic organisms [27]. Salinity affects osmotic regulation or ion concentration modifications and consequently changes the size of cells [28,29]. In addition, it allows for the adjustment of intra/extracellular enzymes and metabolic activities [28]. The effects of salinity on toxin production have been evaluated in some toxic *Alexandrium* [23,30]; however, their toxicity was not consistent and varied depending on test organisms and salinity concentrations. Toxin production in *A. minutum*, for example, tended to be higher at lower salinity [31]. In contrast, various toxin profiles of *A. catenella* were obtained under different salinities, and the toxicity was positively correlated with salt concentration [30]. As such, the toxicity and toxin-producing action of *Alexandrium* under different salt conditions have not yet been elucidated.

The structure of STXs was revealed in the 1980s [32], and approximately 20 years later, the putative genes involved in their biosynthesis were identified from the toxic freshwater cyanobacterium *Cylindrospermopsis raciborskii* [33]. The STX biosynthesis genes exist as a gene cluster (sxt) that encodes 36 genes [18,33]. They are functionally divided into four groups: core genes, tailoring genes, regulators, and transporters [34]. Of these, eight-core genes (*sxtA*, *sxtG*, *sxtB*, *sxtD*, *sxtS*, *sxtU*, *sxtH*/*T*, and *sxtI*) are suspected to be directly involved in STX biosynthesis [33,35]. In toxic dinoflagellates, STX biosynthesis genes were first identified in *Alexandrium fundyense* [13], and these have similar genetic structures to those of toxic cyanobacteria [34,36]. Among these, *sxtA* is the most thoroughly researched gene in *Alexandrium* because it is involved in the first step of STX biosynthesis [13,37]. Recent genomic studies have shown that variations in the sxtA4 domain within the *sxtA* were observed in STX-producing *Alexandrium*, but not in non-producing species [38,39]. Thus, the sxtA4 domain is suspected to play a critical role in STX production, and its expression is associated with toxin synthesis [40]. In addition, the second core gene of STX synthesis is *sxtG*, which is proposed to incorporate an amidino group from the product of *sxtA* [33]. For these reasons, several studies have attempted to evaluate the gene expression profiles of *sxtA4* and *sxtG* in toxic *Alexandrium* under different environmental conditions such as nutrient availability and temperature [25,41,42] but not under different salinities.

In the present study, we assessed the effect of salinity on the cellular physiology, toxin production, and expression of the two STX biosynthesis core genes, *sxtA4* and *sxtG*, in the toxic dinoflagellate *Alexandrium pacificum*. In addition, we compared the relationship between salinity and toxin content through transcriptional regulation. The test species *A. pacificum* (formerly *A. tamarense*) is known to produce STXs [13,24] and is widely distributed in the coastal waters of Europe, Australia, Asia, and America [43,44,45,46,47]. In Korea, the species was first recorded in 1985 [48], and it has been continuously investigated in terms of environmental monitoring and toxicity because of its early spring blooms and toxin outbreaks [49,50].

## 2. Results

### 2.1. Effects of Salinity on Cell Growth and Cell Size

**Figure 1:**
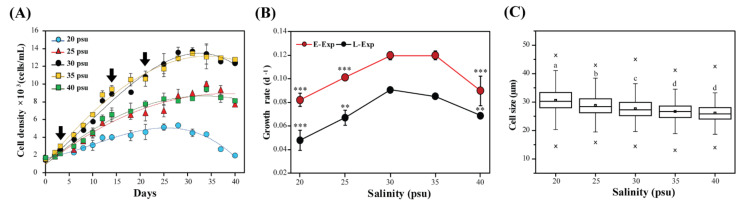
Growth curves of Alexandrium pacificum during the 40 days experimental period (**A**), the growth rate at 14 days (early exponential phase) and 21 days (late exponential phase) (**B**), and box plots of the cell size at 21 days (**C**) under various salinity concentrations (20, 25, 30, 35, and 40 psu). Error bars represent standard deviations. Arrows in (**A**) represent the sampling points (3, 14, and 21 days) for saxitoxins (STXs) analysis. Significant differences among the samples are indicated by different lowercase letters (a, b, c, and d), as determined using a one-way ANOVA, while ** *p* < 0.01 and *** *p* < 0.001. The “×” were determined by the minimum and maximum value of the cell size. □ represents the mean value of the cell sizes.

The Korean *A. pacificum* Alex05 was successively adapted to the different salinity conditions, and its growth was observed over 40 days. The salinity influenced *A. pacificum* growth, with the cell density highest at 30 and 35 psu, followed by 25 and 40 psu, and the lowest at 20 psu (Figure 1A). Similar growth following a sigmoidal pattern was observed at 30 and 35 psu, with the highest cell density at day 31. In contrast, cells did not grow significantly at 20 psu compared with cells from other treatments. Cell density after day 34 decreased in all salinity conditions, possibly entering the death phase.

The growth rates were higher in the early exponential phase than in the late exponential phase at all salinities (Figure 1B). In each phase, the lowest value was observed at 20 psu (0.08 and 0.05 cell division/d for the early and late exponential phases, respectively). It was 1.46-times lower in the early phase and 1.89-times lower in the late exponential phase than that of the control. The growth rates, however, were not significantly different between 30 and 35 psu (*p* > 0.005).

In addition, average cell size gradually decreased with increasing salinity (Figure 1C). The highest size (31.53 ± 4.9 µm) was recorded at 20 psu, while the smallest was 25.85 ± 3.5 µm at 40 psu. The cell sizes from these treatments were significantly different (*p* < 0.001).

### 2.2. Toxin Content and Composition at Different Salinities

**Figure 2:**
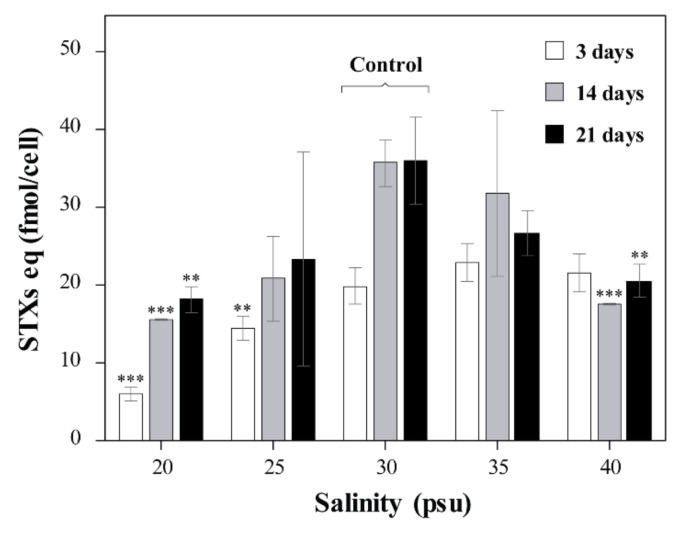
Saxitoxin content (STXs eq) from Alexandrium pacificum Alex05 cultured at various salinity concentrations (20, 25, 30, 35, and 40 psu) after 3, 14, and 21 days. Error bars represent standard deviations. Significant differences between the control (30 psu) and treatments, as determined by one-way ANOVA, are indicated with ** *p* < 0.01 and *** *p* < 0.001.

Total STXs eq of A. pacificum Alex05 varied from 5.98 to 35.82 fmol/cell in our tested salinities (Figure 2). It tended to increase over time in the control group. The toxin contents, however, considerably decreased at 20 and 40 psu in all test periods. There were no significant differences between 25, 30, and 35 psu on days 14 and 21 (*p* > 0.05).

**Figure 3:**
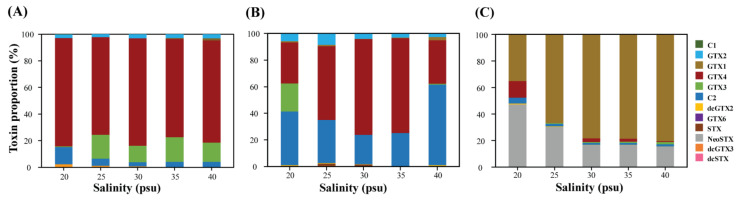
Toxin profiles of *Alexandrium pacificum* after three days (**A**), 14 days (**B**), and 21 days (**C**) of incubation under different salinity concentrations (20, 25, 30, 35, and 40 psu). Different colours represent the different toxin derivatives. STX, saxitoxin; C, N-sulfocarbamoyl; GTX, gonyautoxins; dc, decarbamoylated; NeoSTX, neosaxitoxin.

STX profiles changed depending on the salinity conditions and growth phases (Figure 3). Overall, salinity affected not only the total STXs eq but also the toxin profiles of A. pacificum Alex05. A. pacificum primarily produced GTX4 and GTX3 (total ≥ 81.8%) in the initial stage, which changed to GTX4 and C2 (total ≥ 71.2%) on day 14. In contrast, high proportions of GTX1 (≥35.1%) and NeoSTX (≥15.3%) were detected on day 21. Interestingly, the toxin profile at 20 psu was markedly different from that at other salinities. In particular, GTX3 decreased with an increase in C2 (12.9%) on day 3, whereas high proportions of GTX3 (21.2%) and GTX4 (12.45%) were observed on days 14 and 21, respectively.

### 2.3. Expression Levels of sxtA4 and sxtG in A. pacificum under Different Salinities

**Figure 4:**
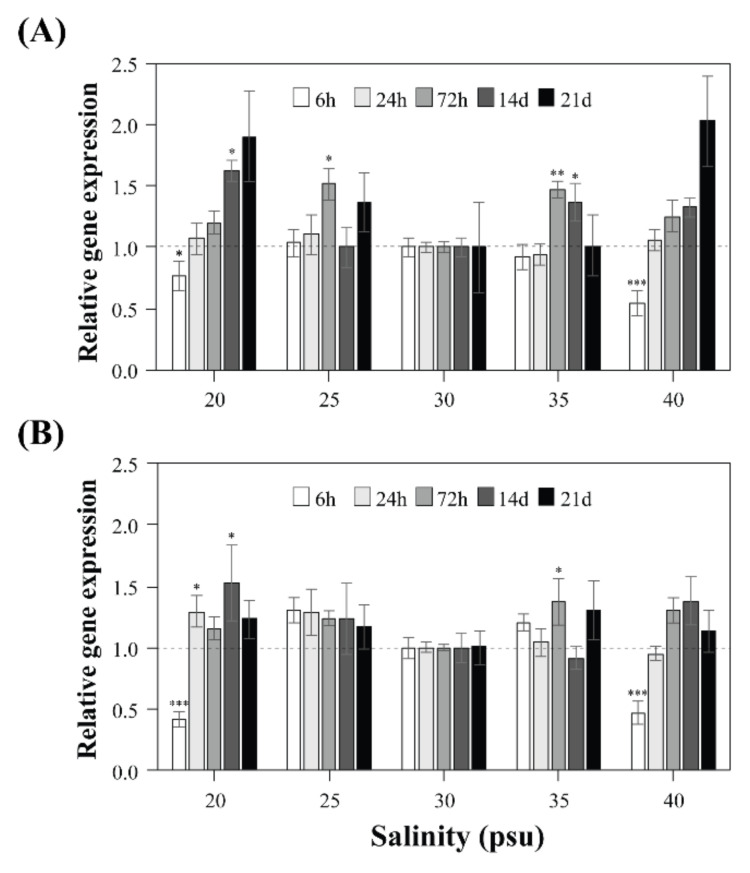
Gene expression levels of *sxtA4* (**A**) and *sxtG* (**B**) in *Alexandrium pacificum* under various salinity concentrations (20, 25, 30, 35, and 40 psu) at different time points. Error bars represent standard deviations. Significant differences between the control (30 psu) and treatments, as determined by a one-way ANOVA, are indicated by * *p* < 0.05 and ** *p* < 0.01.

Gene expression levels of STX synthesis *sxtA4* and *sxtG* were evaluated during the initial stages (6 h, 1 day, and 3 days), the early exponential phase (day 14), and the late exponential phase (day 21) (Figure 4). The relative gene expression was examined at each point by comparing each treatment with 30 psu as a control. Overall, *sxtA4* and *sxtG* expression patterns were not significantly different among all tested salinities, particularly from 25 to 35 psu. Their gene expression, however, responded early in the extreme salinity conditions, and similar patterns for *sxtA4* and *sxtG* were also observed at 20 psu and 40 psu. Specifically, these genes were downregulated at 20 psu (1.3-fold in *sxtA4* and 2.3-fold in *sxtG*) and 40 psu (1.8-fold in *sxtA4* and 2.1-fold in *sxtG*) compared with those of the control after 6 h of incubation, and subsequently, the levels steadily increased over time.

### 2.4. Statistical Correlations among Salinity Concentrations, Gene Expression, and Biological Data

**Figure 5:**
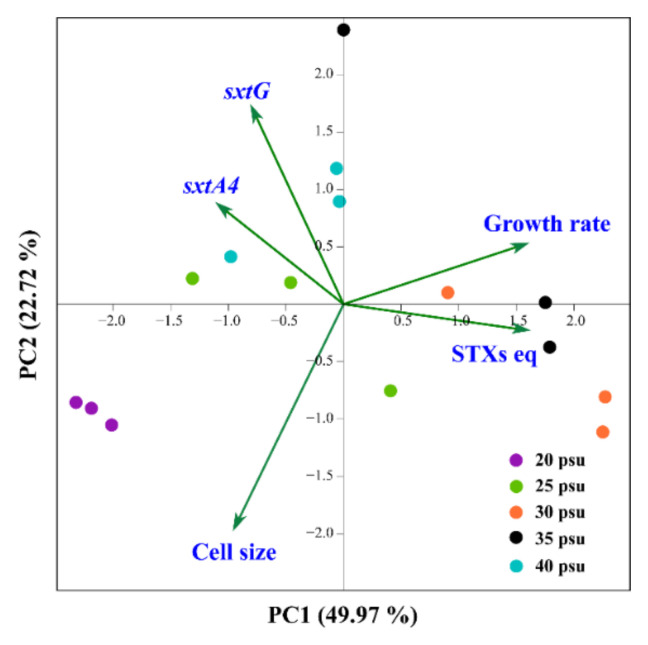
Principal component analysis (PCA) performed with the growth rate, cell size, toxin content (STXs eq), and sxtA4 and sxtG gene expression at various salinity concentrations.

The PCA plot showed relationships among the growth rate, cell size, STXs eq, and gene expression of *sxtA4* and *sxtG* at various salinities (Figure 5). The principal components PC1 and PC2 explained 49.97% and 22.72% of the variance, respectively. The expression levels of the two core genes were clustered together on the negative axis of PC1. In contrast, the total STXs eq and growth correlated with 30 and 35 psu and were positioned on the positive part of the PC1 axis. No relationship was observed between gene expression levels and toxin content. Instead, the low salinity (20 psu) was proportional to cell size.

## 3. Discussion

Toxic *Alexandrium* species are one of the most studied dinoflagellates because of their toxicity and harmful impact on marine environments [5,51]. Their growth and PSP outbreaks vary depending on environmental conditions and genetic traits [1,52,53]. Understanding the influence of environmental factors on STX production can provide a tool for monitoring and early prediction of the occurrence of blooms and PSP incidents [54,55,56]. Although the effects of salinity on STX toxicity of *Alexandrium* species have been studied from a chemical perspective for a long time [30,31,52], the genetic regulation of putative STX biosynthesis genes has not been revealed. The present study is the first evaluation of the effects of salinity on the growth, STX production, and gene expression of *sxtA4* and *sxtG* in a Korean strain of *A. pacificum*.

*Alexandrium* species are known to be rather euryhaline and have different salinity adaptation ranges depending on the species and physiological characteristics (Table 1). Malaysian *Alexandrium minutum*, for example, exhibited strong salinity tolerance (5–30 psu), whereas *A. tamiyavanichii* and *A. tamarense* grew at a salinity range of 20–30 psu. In contrast, Vietnamese *A. minutum* had a salinity range of 10–15 psu [57]. Our study showed that the optimal salinity of Korean *A. pacificum* was 30 and 35 psu. This matched the salinity measured when *A. catenella* and *A. pacificum* were observed in Masan Bay, Korea [58]. However, Korean *A. insuetum* can grow at salinities as low as 15 psu, with a maximum growth rate observed at 25 psu [59]. This suggests that the optimal salinity for cell growth may depend on the species or geographical origins of *Alexandrium* [37]. Even ecotype variations in salinity tolerance may be explained by the growth adaptation of *Alexandrium* species [31,60].

The cell size of our tested *A. pacificum* was significantly higher under low salinity conditions. This is similar to previous results in Malaysian *A. tamiyavanichii*, *A. tamarense*, and *A. peruvianum* [31]. An increase in the cell size was also observed in another dinoflagellate, *Prorocentrum cordatum*, when cultured under low salinity [64]. In contrast, Lim and Ogata found a positive correlation between the cell size and salinity conditions in *A. minutum* [31]. Although the responses to salinity of *Alexandrium* species are inconsistent, cell size should be related to their ability to regulate osmolality. When the salinity changes, the cell membrane must constantly adjust the cell volume to maintain equilibrium between intracellular and extracellular osmosis and achieve a stable environment for optimal metabolic function [65]. In addition, under high salinity, the surface area/volume ratio may increase by reducing the cell size, which makes diffusion or osmosis much more effective [31]. This osmotic action can explain the negative correlation between the *A. pacificum* cell size and salt concentration.

To date, several studies have revealed the relationship between growth and toxicity of *Alexandrium* depending on the growth stage and culture conditions [30,31,66]; however, their results are not consistent. Indeed, the toxicities and growth rates of both *A. catenella* and *A. tamiyavanichii* were positively correlated in salinity experiments [30,31]. This pattern is in accordance with our results for *A. pacificum*. However, it was inversely related in *A. tamarense* and *A. minutum*, and no correlation was observed in *A. peruvianum* [22,31]. Based on these results, the difference in toxicity cannot be clearly explained, but it is presumed to be an effect of salinity-induced metabolism.

Changes in the membrane transport processes are one of the first cell responses to variations in salinity [27], which cause modulations in metabolic functions such as arginine synthesis [67]. Such salinity stress can reduce the activities of ornithine, glutamine, and carbamyl phosphate synthesis, thereby decreasing arginine metabolism [68]. Since arginine is a precursor of STX biosynthesis [32], extreme salinity conditions (e.g., 20 and 40 psu) may suppress STX production by toxic dinoflagellates by affecting arginine synthesis [31].

The strength of STX toxicity differs depending on the toxin content and profiles in *Alexandrium*. Previous studies and the present results detected different STX profiles in *Alexandrium* under different salinity conditions [23,37], possibly altering their toxicity. For example, the percentages of GTX2/3 and STX in *A. ostenfeldii* and *A. tamarense* decreased under higher salinity [37]. In addition, *A. ostenfeldii* produced more STX and less C1/2 under low salinity conditions [60]. In the present study, we also observed that the toxin profiles of *A. pacificum* Alex05 changed depending on the salinity conditions and growth stage. Overall, β-epimers of 11-hydroxysulfate toxins (GTX3/4, C2, etc.) were primarily detected on days 3 and 14, and they were changed to α-epimers of 11-hydroxysulfates (GTX1/2, C1/3, etc.) and NeoSTX on day 21. In contrast, our *A. pacificum* tended to produce more C-toxins under extreme salinity conditions (20 and 40 psu) on days 3 and 14 compared with the optimal salinity conditions. The strength of STX analogue toxicity decreased in the order of STX → NeoSTX → GTX4/3 → C1/2; thus, changes in their levels can explain the lower toxicity of *A. pacificum* at 20 and 40 psu [69,70]. On day 21, the NeoSTX ratio at 20 and 25 psu was higher than that in the other samples; however, the lower concentrations resulted in a lower total STXs eq. These findings and our results suggest that changes in salinity may affect STX and analogue production in toxic *Alexandrium*.

The toxin composition and toxicity should be related to the metabolic pathway, in which STX biosynthesis enzymes are involved [11]. They are encoded in the nuclear genome; thus, the presence or absence of toxins may provide evidence for genetic differences [38,71]. In addition, many studies have reported that toxin profiles may be regulated by toxin-related gene expression under various environmental conditions, such as temperature and nutrient availability [25,42]. Our previous research showed that toxin content and *sxtA4* and *sxtG* expression levels were considerably increased in *A. pacificum* and *A. catenella* under low temperatures and cold shock treatments [24,42]. In other studies, STX production under different culture conditions was independent of the transcriptional levels of the core *sxt* genes [41,72]. The *sxtA* expression level of the cyanobacterium *Aphanizomenon gracile* was upregulated at extreme temperatures, but this did not correlate with STX concentration [72]. In addition, *sxtA1* and *sxtG* expression in the dinoflagellate *A. minutum* did not reflect STX accumulation under nutrient limitation [41]. These results were similar to our results in that the transcriptional levels of *sxtA4* and *sxtG* did not statistically match with the total STXs eq in *A. pacificum*. This implies that salinity affects STX production; however, STX production may not be regulated by enzymes *sxtA* and *sxtG*, because they are involved in the early stages of STX synthesis. Instead, the changes in STX profiles under different salinities may be caused by other genes acting in the late stages of toxin modification [17,18,72].

As noted previously, when STX and its analogues are synthesised, many enzymatic reactions such as toxin biosynthesis, tailoring, regulators, and transporters are involved [33]. In particular, since STX toxicity differs depending on the analogue composition and/or content, the enzymes involved in toxin modification may play a crucial role in determining toxicity. In this context, certain STX tailoring genes (e.g., *sxtL*, *sxtN*, *sxtO*, *sxtX*, etc.) seem to be involved in the toxicity difference under salinity changes [18]. This possibility was supported by the enzymatic functions of GTXs produced by mono-sulfation of STX at the C11 position via an O-sulfotransferase that is encoded by tailoring the *sxtL* gene [18]. In addition, N-sulfotransferase (*sxtN*) is responsible for the N-sulfation of STX to produce C-toxins [17]; *sxtX* encodes a cephalosporin hydroxylase enzyme, which produces N1-hydroxylated analogues of STX, like NeoSTX [33]. When considering these findings, the effect of salinity on the STX profiles that may result from the regulation of tailoring genes and enzymes needs to be accounted for.

## 4. Conclusions

In the present study, we revealed the correlation between growth rate, cell size, and toxin production in toxic *A. pacificum* under different salinities. The STX toxicity was highest at the optimal salinity, and the toxin composition varied considerably in different salinities and growth stages. The gene expression levels of *sxtA4* and *sxtG* decreased under extreme salinities; however, there were no significant relationships between gene expression and STX toxicity in *A. pacificum*. Based on these results, we can conclude that the salinity alters STX content and profiles, but the two core genes of STX synthesis, *sxtA4,* and *sxtG*, may not be directly involved. Other STX tailoring genes and enzymes are possibly involved, and thus future studies should evaluate the responses of these genes in toxic *Alexandrium* under different salinities.

## 5. Materials and Methods

### 5.1. Strain and Culture Conditions

The strain Alex05 (formerly LMBE-C4) of toxic *Alexandrium pacificum* was obtained from the Marine Bio Resource Information System (MBRIS) at the Korea Institute of Ocean Science and Technology (KIOST, Jangmok, Korea). It was cultured in f/2 medium without silicate [73] at 16 °C under a 12:12 h light:dark cycle with approximately 65 μmol photons/m2/s.

### 5.2. Salinity Experiment Setup

The effects of salinity on the growth and toxin production of *A. pacificum* were tested at 20, 25, 30 (control), 35, and 40 psu. These salinity ranges were chosen by considering the tolerance data recorded from field surveys of *Alexandrium* spp. in Korean coastal waters [74,75]. Each salinity condition was adjusted by adding distilled water or by evaporating filtered seawater in a drying oven at 60 °C. Changes in salinity were verified using the handheld refractometer JA 100 (Jaco, Seoul, Korea). To minimise the shock caused by rapid changes in salinity, cultures were pre-acclimated to the respective experimental conditions according to Yamamoto et al. [76]. In particular, stock cultures were inoculated into the salinity-modified f/2 medium step-by-step, with each step acclimated after four days.

For the salinity experiment, we prepared five flasks containing 600 mL of salinity-modified f/2 medium with 20, 25, 30, 35, and 40 psu. The experiment was started by inoculating 300 mL of the five-salinity-acclimated stock cultures into individual flasks. The salinity concentrations were re-checked using the handheld refractometer. All samples were performed in duplicate with the same initial cell density of 1.5 × 10^3^ cells/mL.

### 5.3. Cell Growth and Cell Size Measurements

Growth patterns were monitored by cell density and determined after 40 days. Cultures were sampled and fixed with Lugol fixative reagent (final concentration 1%) for cell counting. Cell density was determined using a plankton-counting chamber (Matsunami Glass, Osaka, Japan) under a light microscope (Carl Zeiss Axioskop, Oberkochen, Germany). The growth rate was calculated with cell counts using the equation: µ = (lnN2 − lnN1)/(t2 − t1), where N1 and N2 are the average values of the cell numbers at times t1 and t2, respectively.

To measure cell size, 20 µL of the cell suspension was collected after centrifuging 2 mL of culture from each salinity condition. These were pipetted into a Cellometer disposable cell counting chamber (Nexcelom Biosciences, Lawrence, MA, USA). The cell size was automatically analysed using a Cellometer Auto T4 (Nexcelom Biosciences, Lawrence, MA, USA).

### 5.4. STX Analysis

To determine the toxin content, we harvested 100 mL of culture at the tested salinity concentrations and centrifuged these samples at 10,000 rpm for 10 min. Pellets were collected and mixed with 1 mL of 0.01 M HCl (pH 3.0), and then homogenised using a bead beater (BioSpec Products Inc., Bartlesville, OK, USA) with zirconium beads (0.1 mm in diameter). After boiling at 95 °C for 5 min, the samples were filtered using a 0.2 µm GVS syringe filter (GVS, Bologna, Italy). Toxin analysis was performed via high-pressure liquid chromatography-fluorescence detection (HPLC-FLD) (Waters, Milford, MA, USA) using the post-column reaction method following Rey et al. [77], with slight modifications. In detail, the flow rate of binary pump (Waters, Milford, MA, USA) was 0.3 mL/min with solvent A: 0.075% (*v*/*v*) trifluoroacetic acid, and solvent B: 0.025% (*v*/*v*) trifluoroacetic acid. The gradient method was started with 4% solvent B, 4–25% solvent B in 50 min, back to 4% solvent B at 50.01 min, and kept the flow for 10 min before the next injection. In this analysis, we used twelve standard solutions of STXs, including C1-2, dcGTX2-3, dcSTX, GTX1-4,6, NeoSTX, and STX, that were obtained commercially from the National Research Council Canada (NRC, Ottawa, Ontario, Canada). Reference curves of each standard were constructed by measuring six known concentrations (R^2^ ≥ 0.98). Limits of detection (LOD) and quantification (LOQ) for the standards were estimated to be 0.32 and 0.97 ng/mL, respectively. Tested samples and 12 standards were separated using a 5 µm Hypercarb^®^ column (150 mm × 2.1 mm internal diameter; Thermo Scientific, Madrid, Spain) with different retention times and oxidised under isocratic conditions. For the specific oxidisation, the flow rate of oxidant (100 mM H_3_PO_4_, 10 mM H_5_IO_6_, adjusted at pH 7.8 with 10 M NaOH) and acid (0.1 M HNO_3_) were set at 0.5 mL/min and 0.3 mL/min, respectively. The reaction was induced by setting the temperature of the post-column reaction module at 85 °C. The standards and samples were monitored using a fluorescence detector at 330 nm for excitation and 390 nm for emission wavelengths.

### 5.5. RNA Extraction and cDNA Synthesis

Total mRNA was extracted from 200 mL of *A. pacificum* cultures at different time points. Cells were harvested and centrifuged at 3500 rpm for 5 min at 4 °C, frozen immediately in liquid nitrogen, and stored at −80 °C until RNA extraction. These were physically broken by freeze-thawing in liquid nitrogen, and further homogenisation was performed using a mini-bead beater (BioSpec Products Inc., Bartlesville, OK, USA). The total RNA was isolated using TRIzol reagent (Invitrogen, Carlsbad, CA, USA) and further purified using the Mini Spin Columns of Rneasy Mini Kit (Qiagen, Valencia, CA, USA). The RNA quality and quantity were measured using a Nanoready Micro UV-Vis spectrophotometer (Hangzhou Lifereal Biotechnology Co., Ltd., Hangzhou, Zhejiang, China). Reverse transcription was performed using a TOPscriptTM cDNA Synthesis Kit (Enzynomics, Daejeon, Korea) for gene expression experiments.

### 5.6. Quantitative Real-Time PCR

Quantitative real-time PCR (qRT-PCR) reactions were carried out using the primers *sxtA4* (ApsxtA-F, 5′-TGGTGATCTACGGTCAGAGG-3′; ApsxtA-R, 5′- GGCATCATGTA CTGGAAGCAC-3′) and *sxtG* (ApsxtG-F, 5′- CATCCCAGACTGGTACATGC-3′; ApsxtG -R, 5′-GGATGTACCTGTGCATCTCG-3′), following the previous study [24]. The qPCR assays were performed using the TOPrealTM qPCR 2 × PreMIX SYBR Green Kit (Enzynomics Inc., Daejeon, Korea) in a CFX96 Real-time PCR Detection System (Bio-Rad, Hercules, CA, USA). Reaction mixtures were prepared as follows: 1 µL each of the forward and reverse primers (10 pmol/µL), 2 µL of cDNA, 10 µL of TOPreal qPCR 2 × PreMIX buffer (SYBR Green with high ROX), and 6 µL distilled water. The reaction was performed as follows: 50 °C for 4 min; 95 °C for 10 min; and 40 × (95 °C for 10 s; 60 °C for 15 s; 72 °C for 15 s). All samples were run in triplicate to calculate the mean value. The reference genes actin (ApACT-F, 5′-TTCCGGCGATGTACGTTG-3′; ApACT-R, 5′-TAGGCAC AGTGTGTGACACGC-3′) and β-tubulin (ApTUB-F, 5′-ACGTTCTCGGTGATCCCAT-3′; ApTUB-R, 5′-AGCGTGCGGAAACAGATG-3′) were used as internal controls for data normalisation [24].

### 5.7. Statistical and Principal Component Analysis

All data are presented as the mean ± standard error. Significant differences were analysed by One-Way ANOVA using SPSS software ver. 20 (IBM Corp., Armonk, NY, USA). The probability (*p*) values of One-Way ANOVA were indicated as * *p* < 0.05, ** *p* < 0.01, and *** *p* < 0.001. The cell size data were statistically calculated using Origin ver. 8.5 (OriginLab Corp., Northampton, MA, USA).

Principal component analysis (PCA) was used to examine the relationships between salinity parameters and the other biological data, including growth rate, cell size, STXs equivalent (STXs eq), and relative gene expression of *sxtA4* and *sxtG*. The PCA plot was constructed using the Palaeontological Statistics Package Past ver. 4.03 [78].

## Figures and Tables

**Table 1:** Optimal growth condition and toxin production in the genus *Alexandrium* under different salinity from published literature.

Species	Strain	Salinity Range	Optimal Growth Salinity	Highest Toxin Condition	Toxins	STXs Eq (fmol/cell)	Reference
*A. catenella*	PFB38	15–35	35	35	NeoSTX, GTX1-5	95.76	[61]
	ACT03	10–40	30	35	C1-4, GTX3-5	50.3	[30]
*A. fundyense*	MI	15–35	25	30	STX, NeoSTX, GTX1-4	62	[21]
	BoF	15–35	25	30–35	STX, NeoSTX, GTX1-4	73–75	[21]
*A. minutum*	AM89BM	12–37	20–37	15	-	50	[22]
	AmKB06	2–30	15	5	GTX1-6, C2, NeoSTX, dcSTX	12	[31]
	Alexsp17	5–35	10–15	30–35	STX, NeoSTX, dcSTX, C2, GTX2-4, GTX4-12ol	30	[57]
*A. ostenfeldii*	AOSH1	15–33	33	15	C3, C, desmthyl D	-	[62]
	OKNL21	3–34	22	5	STX, GTX2/3/5, C1-2	52	[63]
*A. peruvianum*	ApKS01	2–30	25	25	GTX1-6, C2, NeoSTX, dcSTX	0.75	[31]
*A. tamarense*	Pr18b	10–30	25‰	25	STX, NeoSTX, GTX1-4, C1-3	179	[23]
	AtPA01	2–30	20–30	15	GTX1-6, C2, NeoSTX, dcSTX	0.8	[31]
*A. tamiyavanichii*	AcMS01	2–30	25	20	GTX1-6, C2, NeoSTX, dcSTX	80	[31]

- No data.

## Data Availability

The data presented in this study are available in this article.
